# Supplementary material for: Splice-Junction-Based Mapping of Alternative Isoforms in the Human Proteome
Source: Cell Rep. Author manuscript; Available in PMC 2020 Jan 15. (PMC6961840; doi:10.1016/j.celrep.2019.11.026)
Supplement: 2 [file NIHMS1546469-supplement-2.zip › DF1/PXD009737/TestisLysC/TestisLysC_36_RANBP3_GPAIAPPVFVFQK.pdf]

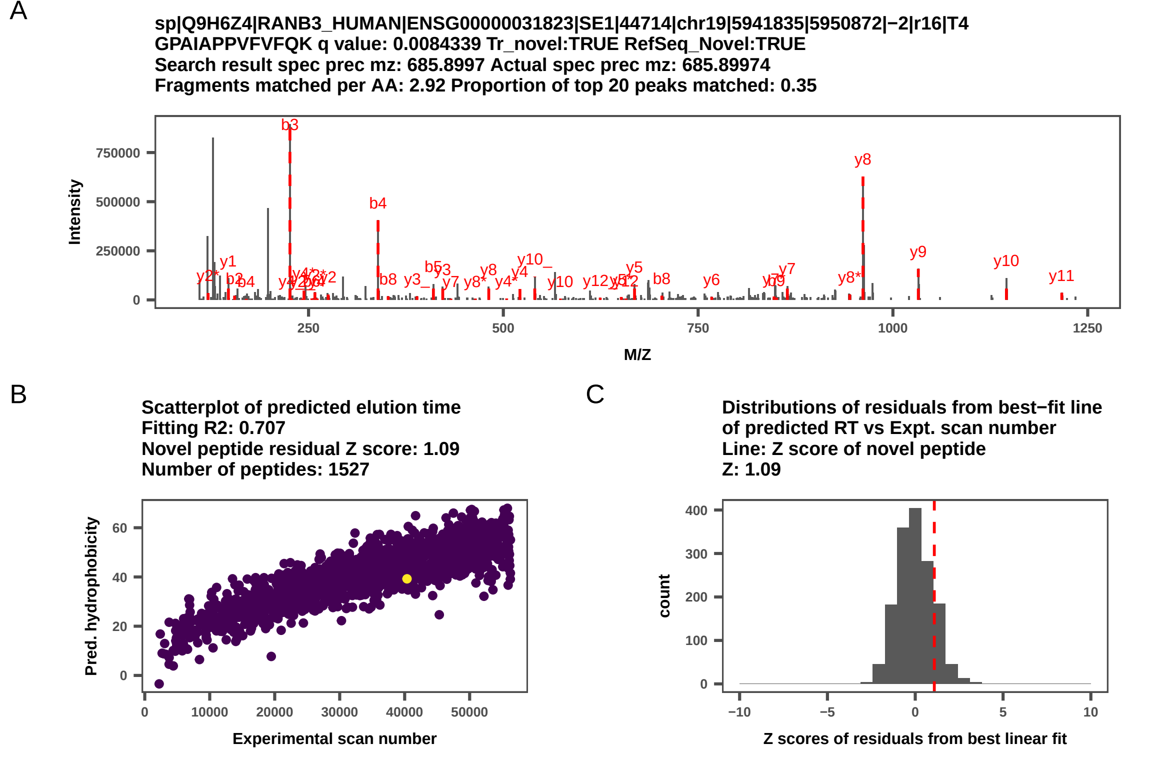
A
sp|Q9H6Z4|RANB3_HUMAN|ENSG00000031823|SE1|44714|chr19|5941835|5950872|−2|r16|T4
GPAIAPPVFVFQK q value: 0.0084339 Tr_novel:TRUE RefSeq_Novel:TRUE
Search result spec prec mz: 685.8997 Actual spec prec mz: 685.89974
Fragments matched per AA: 2.92 Proportion of top 20 peaks matched: 0.35
Intensity
750000
500000
250000
0
250
500
750
1000
1250
M/Z
b3
b4
y8
y9
y10
y11
y1
b8
y6
y7
y8*
y5
B
Scatterplot of predicted elution time
Fitting R2: 0.707
Novel peptide residual Z score: 1.09
Number of peptides: 1527
Pred. hydrophobicity
60
40
20
0
0
10000
20000
30000
40000
50000
Experimental scan number
C
Distributions of residuals from best−fit line
of predicted RT vs Expt. scan number
Line: Z score of novel peptide
Z: 1.09
count
400
300
200
100
0
−10
−5
0
5
10
Z scores of residuals from best linear fit
